# Development of a Wearable Glove System with Multiple Sensors for Hand Kinematics Assessment

**DOI:** 10.3390/mi12040362

**Published:** 2021-03-27

**Authors:** Fei Fei, Sifan Xian, Xiaojian Xie, Changcheng Wu, Dehua Yang, Kuiying Yin, Guanglie Zhang

**Affiliations:** 1 College of Automation Engineering, Nanjing University of Aeronautics and Astronautics, Nanjing 211100, China; sifanxian@nuaa.edu.cn (S.X.); xie_xiaojian@163.com (X.X.); changchengwu@nuaa.edu.cn (C.W.); dhyang@nuaa.edu.cn (D.Y.); 2 Nanjing Research Institute of Electronic Technology, Nanjing 210013, China; yinkuiying@cetc.com; 3 Department of Mechanical Engineering, City University of Hong Kong, Hong Kong 999077, China; guanglie.zhang@gmail.com

**Keywords:** hand rehabilitation, data glove, inertial sensors, kinematics evaluation

## Abstract

In traditional hand function assessment, patients and physicians always need to accomplish complex activities and rating tasks. This paper proposes a novel wearable glove system for hand function assessment. A sensing system consisting of 12 nine-axis inertial and magnetic unit (IMMU) sensors is used to obtain the acceleration, angular velocity, and geomagnetic orientation of human hand movements. A complementary filter algorithm is applied to calculate the angles of joints after sensor calibration. A virtual hand model is also developed to map with the glove system in the Unity platform. The experimental results show that this glove system can capture and reproduce human hand motions with high accuracy. This smart glove system is expected to reduce the complexity and time consumption of hand kinematics assessment.

## 1. Introduction

Hand function assessment plays an essential role in the recovery of stroke patients. Rehabilitation of hand function always requires long-term training after medical treatment. Traditional hand function assessment methods that have been used by physicians include Fugl-Meyer assessment (FMA), the action research arm test (ARAT), Jebsen–Taylor test (JTT), or Wolf motor function test (WMFT) [1,2,3,4,5]. With these methods, the patients must carry out some prescribed actions to showcase their hand function, and rating scores are given according to the patients’ performance. However, assessment with these methods often takes several hours to complete and does not reveal enough details during the rehabilitation process. Therefore, it is important to develop hand function assessment equipment that can help physicians make a quantitative analysis of patients’ rehabilitation status. 

One obvious solution to get hand gesture information is using a visual camera system [6,7,8]. The advantage of using such a system is that it does not require the patients to wear any equipment and it can capture the gesture information of multiple patients at the same time. The disadvantage is that it can only work effectively in good light conditions and its shooting angle is highly restricted by the local environment. IR or radar sensors can be another non-contact alternative for hand gesture capture or recognition [9,10]. 

In recent years, data gloves have been widely used in human–computer interaction studies [11]. With the advancement of sensor technology, the cost of data gloves has been greatly reduced to suit daily medical rehabilitation needs [12]. Depending on their working mechanism, data gloves can generally be divided into these main types: mechanical, flexible sensing, optical fiber sensing, and inertial sensing. A mechanical data glove can be considered as a type of glove-like haptic device with force feedback. Blake et al. proposed a haptic glove for virtual reality applications based on magnetorheological (MR) brakes in 2009 [13]. Ma et al. developed a five-finger haptic glove using a worm-geared motor in 2015 [14]. Chiri et al. developed a multi-phalange finger module for post-stroke rehabilitation in 2012 [15]. Gu et al. presented a low-cost exoskeleton glove with passive force feedback in 2016 [16]. Unlike that of mechanical data gloves, the performance of flexible sensing data gloves largely depends on the repeatability and linearity of the sensor itself. Kostas et al. presented a data glove with flexible sensors early in 2003 [17]. Togetti et al. presented a sensing glove made of conductive elastomer materials in 2006 [18]. Shen et al. also developed a soft stretchable bending data glove that incorporates a sensor based on ethylene propylene rubber (EPR) in 2016 [19]. However, the most successful commercial data glove systems today are mainly based on optical fiber sensors, such as CyberGlove and 5DT data gloves. Innovations have also occurred in the research community for the design of optical fiber data gloves. Fujiwara et al. discussed a low-cost and flexible optical fiber glove to measure joint angles in 2014 [20]. Da Silva et al. developed a sensing glove based on optical fiber Bragg grating (FBG) sensors in 2011 [21]. Although optical fiber-based data gloves such as CyberGlove and 5DT data gloves can provide sufficiently precise joint angle data for rehabilitation applications, they are still too expensive for daily rehabilitation at home. Low-cost MEMS-based inertial sensors have been investigated in a number of studies on data gloves or human interactions due to their ability to provide stable attitudes in a three-dimensional (3D) space and ease of integration with PCBs. Lin et al. presented a data glove system with six-axis inertial sensors for stroke evaluation [22,23]. Choi et al. developed a low-cost inertial measurement unit (IMU) wearable sensing glove [24]. Liu et al. designed a novel inertial and magnetic unit (IMMU)-based data glove with an optimized sensor layout [25]. 

The data glove system discussed in this paper aims to help physicians evaluate patients’ hand function during rehabilitation. To get the joint angles of hand attitudes, 12 nine-axis inertial sensors are integrated with the Micro Controller Unit (MCU) and Universal Asynchronous Receiver/Transmitter (UART) modules on the glove. Several calibration algorithms are implemented to avoid errors caused by the accelerometer, gyroscope, and magnetometer. In addition, a human–computer interaction module is developed in the Unity platform. A grasping ball experiment is conducted to demonstrate how the glove system can be used to evaluate dynamic hand functions. 

## 2. System Architecture

**Figure 1:**
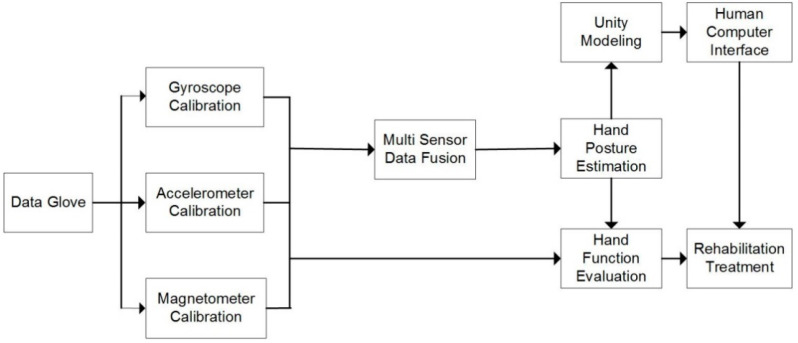
Workflow of the proposed data glove system for hand function evaluation.

The workflow of our data glove system is demonstrated in Figure 1. First, the sensor units installed on the data glove obtain the raw information of hand movements. Then, the MCU controller initializes the data acquisition process, in which the sensor data are received with a Serial Peripheral Interface (SPI) bus and transmitted to the computer through a USB connection line or a Bluetooth transmitter. Finally, the data processing module verifies the data frames and calculates the angles of joints for hand function evaluation. Moreover, the human–computer interaction module uses the joint angle data to drive hand modeling in the Unity platform. 

### 2.1. Hardware Design

**Figure 2:**
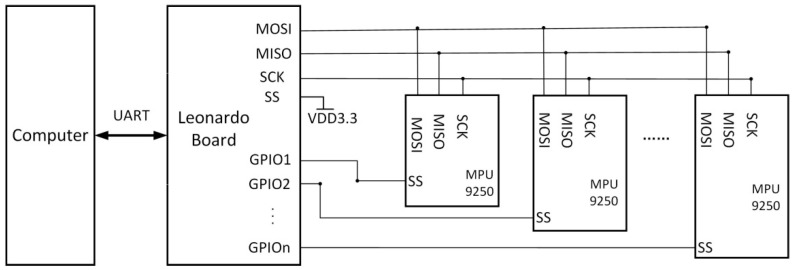
Hardware design of the proposed data glove system.

The hardware of our data glove system includes several parts: (1) some IMMUs for capturing the angles of hand joints; (2) a microcontrol unit for data collection and transmission; (3) a USB connection wire or wireless Bluetooth transmitter; and (4) a PC or laptop for receiving sensor data and reconstructing hand motions. The MEMS-based sensors are uniquely suited for wearable applications due to their advantages of low power consumption, compactness, and high precision. MPU-9250 units from InvenSense, Inc. are used to capture the angles of joints when hand exercises are done. The nine-axis sensor MPU-9250 can be considered as a combination of a six-axis IMU and a three-axis compass, one is the six-axis IMU MPU6515, the other is the AK8963 3-axis magnetometer from Asahi Kasei Microdevices (AKM). The angular velocity range of the gyroscope is set to ±1000°/s and the acceleration range of the accelerometer is set to ±8 g. The sample rate is set to 100 Hz for all the sensors, which is high enough to capture most human hand motions. To achieve better fusion performance, a multi-sensor fusion algorithm is developed to work with the onboard motion fusion processor that comes with MPU-9250. A Leonardo microprocessor chip is used to collect data from the IMMUs. The microprocessor controller has 21 in–out digital ports, 7 Pulse Width Modulation (PWM) ports and 6 analog input ports to connect with 12 sensors via an SPI bus, as shown in Figure 2. Both the wired UART connection and Bluetooth wireless connection are tested in our glove system. The results show that the wired UART connection is more efficient and reliable than Bluetooth wireless connection, especially at a high transmission rate.

### 2.2. Analysis of Hand Joints and Layout of Sensors

**Figure 3:**
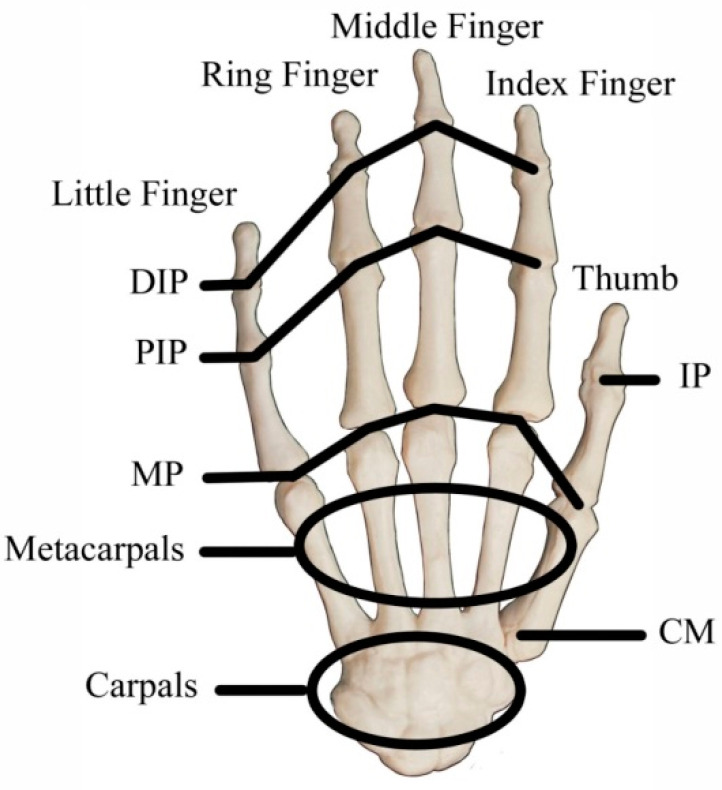
Structure of hand bones and joints.

**Figure 4:**
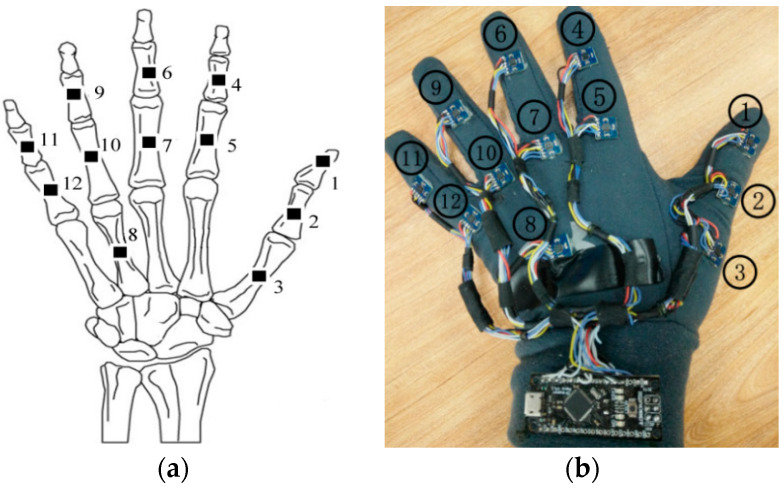
The 1st generation data glove; (**a**) layout of inertial and magnetic unit (IMMU) sensors; (**b**) prototype connected with soft wires.

Human hand motions can be represented by the rotation of hand joints, so the number and position of IMMU sensors can be determined according to the anatomical structures of the hand. A human hand includes several types of bones and tissues such as muscles and ligaments. As shown in Figure 3, the joints of the index finger, middle finger, ring finger, and little finger of a human hand can be divided into three main categories: distal interphalangeal joint (DIP), proximal interphalangeal joint (PIP), and metacarpophalangeal joint (MP). As for the thumb, it only has two joints: interphalangeal joint (IP) and MP. The MP is connected to the carpometacarpal joint (CM) for hand rotation. IMMU sensors should be placed near the abovementioned joints to measure the bending status of fingers. An understanding of the parent–child relationship between joints is a prerequisite for analyzing finger motions. The movement of the parent joints has a knock-on effect on all the child joints. The local x–y–z coordinates should be established to describe the rotation of each joint, as shown in Figure 4. The bending of a finger can be defined as a rotation around the *x*-axis. Empirically, the bending angle of the DIP joint can be approximately considered as 1/3 of the bending angle of the PIP joint, which has no obvious influence on the reconstruction of hand gestures.

**Figure 5:**
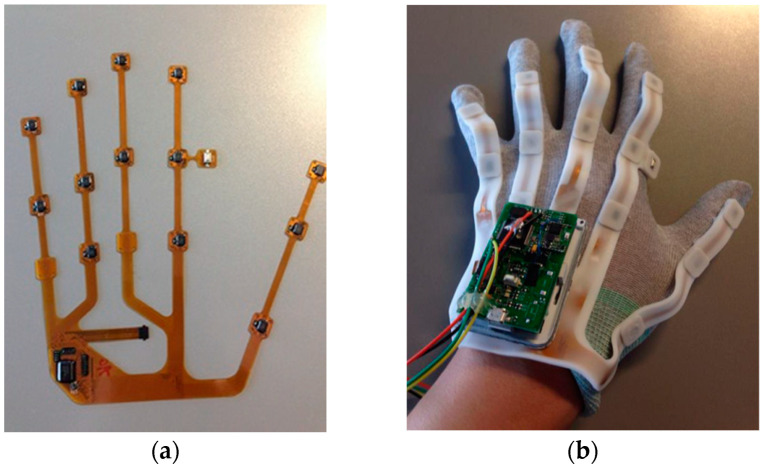
The 2nd generation data glove. (**a**) Flexible PCB and mounted IMMU sensors; (**b**) prototype coated with a silicone protective layer.

Therefore, 12 IMMU sensors are placed on hand phalanges and one IMMU sensor is placed on the back of the hand, as shown in Figure 4. Joint angles and hand gestures can be estimated by the orientation of 11 IMMU sensors. Two generations of data gloves have been developed in our lab. For the 1st generation glove, all the sensors are connected by soft wires; it is easy to fabricate but may suffer from sealing off and bad contact problems. For the 2nd generation data glove, a flexible PCB design is adopted to enhance the system’s stability and reliability, as shown in Figure 5. The IMMU sensors are directly soldered on the flexible PCB without using any wire. Moreover, the flexible PCB is coated with a silicone layer to protect the electronic components from being corroded by moisture and sweat. The thickness of the silicone layer is set to 2 mm so as not to impair the flexibility of hand movements. The plug-in connector of the flexible PCB can be easily linked to a controller board. In this paper, the 1st generation data glove is used to demonstrate the feasibility of our algorithm and software.

## 3. Sensor Data Processing

**Figure 6:**
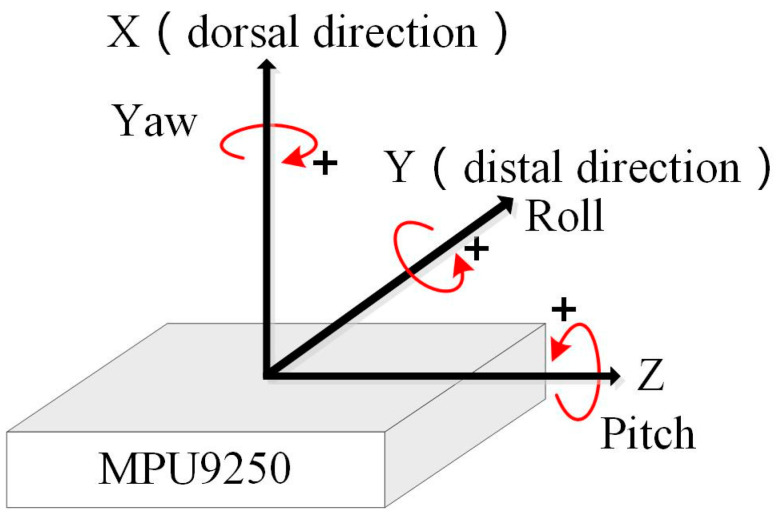
Definition of the orientation of IMMU sensors on the glove of the left hand.

The data processing is as follows: The microcontroller of the data glove sends the data from IMMU sensors to the computer frame by frame, and then the received data are preprocessed by parity frame check and frame decoding. The raw sensor data must be calibrated before they can be fused. Since the environmental influence on accelerometers and gyroscopes is mostly negligible, the calibration parameters of the accelerometer and gyroscope can be used for a long time after initial calibration. Unlike the accelerometer and gyroscope, the magnetometer needs to be recalibrated because it is subjected to the disturbance from environmental ferromagnetic materials. The attitude of each IMMU sensor can be estimated with a multi-sensor fusion algorithm using calibrated acceleration, angular velocity, and geomagnetism data. The orientation of the IMMU sensors on the glove of left hand is as defined in Figure 6 with the International Society of Biomechanics (ISB) recommendations on definitions of joint coordinates [26].

### 3.1. Sensor Calibration

Gyroscopes can be used to measure the rotation of hand joints with angular velocity. The angle of joint rotation can be calculated by integrating the angular velocity data over time. However, the error of angular velocity will accumulate after a long measurement process, resulting in a drift in the angle. Theoretically, the output value of a gyroscope should be zero after calibration without any motions. The zero deviation caused by the random error of the gyroscope can be estimated using the average filtering method. The corrected value of each axis can be obtained by subtracting the zero deviation error from the measured value. The error model of the gyroscope can be represented as follows:

(1) $$\left[\begin{array}{c} g_{x\_c}\\ g_{y\_c}\\ g_{z\_c} \end{array}\right]=\left[\begin{array}{c} g_{x}\\ g_{y}\\ g_{z} \end{array}\right]-\left[\begin{array}{c} g_{x\_e}\\ g_{y\_e}\\ g_{z\_e} \end{array}\right]$$

where $g_{x}$, $g_{y},$ and $g_{z}$ denote the raw data of the gyroscope; $g_{x\_c}$, $g_{y\_c}$, and $g_{z\_c}$ the corrected angular velocity data; $g_{x\_e}$, $g_{y\_e},\;\mathrm{and}\;g_{z\_e}$ the zero bias of three axes. 

Possible errors of the accelerometer include zero deviation error, scale factor error, and non-orthogonal error. The accelerometer error model can be represented as follows [27]:

(2) $$\left[\begin{array}{c} a_{x\_c}\\ a_{y\_c}\\ a_{z\_c} \end{array}\right]=\left[\begin{array}{ccc} m_{xx} & m_{xy} & m_{xz}\\ m_{xy} & m_{yy} & m_{yz}\\ m_{xz} & m_{yz} & m_{zz} \end{array}\right]\cdot \left[\begin{array}{c} a_{x}-a_{x\_e}\\ a_{y}-a_{y\_e}\\ a_{z}-a_{z\_e} \end{array}\right]$$

where $a_{i}\left(i=x,y,z\right)$ denotes the raw data of the accelerometer; $a_{i\_c}\left(i=x,y,z\right)$ the corrected acceleration data; $b_{i}\left(i=x,y,z\right)$ the zero bias; $m_{ij}\left(i=x,y,z;i=j\right)$ the scale error factor; $m_{ij}\left(i=x,y,z;j=x,y,z;i\neq j\right)$ the non-orthogonal error factor. The calibration error $e\left(m_{xx},\ldots ,m_{zz},a_{x_{e}},\ldots ,a_{z\_e}\right)$ is defined as the residual between the squared sum of corrected acceleration and squared gravitational acceleration:

(3) $$e(m_{xx},\ldots ,m_{zz},a_{x\_e},\ldots ,a_{z\_e})=a_{x\_c}^{2}+a_{y\_c}^{2}+a_{z\_c}^{2}-g^{2}$$

Gauss–Newton’s method is applied to estimate the unknown calibration parameters ($m_{xx},\ldots ,m_{zz},a_{x\_e},\ldots ,a_{z\_e}$) in this part. The iteration of calibration error can be represented by the following equation:

(4) $$e_{k+1}=e_{k}+\beta_{k}d_{k}$$

where $\beta_{k}$ is the damping control factor, which is used to control the convergence speed of the iteration algorithm. A larger $\beta_{k}$ value means a faster convergence speed but lower accuracy. $d_{k}$ is defined as the iterative direction:

(5) $$d_{k}={\left(J^{T}J\right)}^{-1}\left(J^{T}\left(-e\right)\right)$$

where matrix *J* is defined as the Jacobian matrix of calibration error:

(6) $$J\left(e\right)=\frac{\partial e}{\partial x}=\left[\begin{array}{ccc} \frac{\partial e}{\partial m_{xx}} & \cdots & \frac{\partial e}{\partial b_{z}}\\ \vdots & \ddots & \vdots \\ \frac{\partial e}{\partial m_{xx}} & \cdots & \frac{\partial e}{\partial b_{z}} \end{array}\right]$$

When the iteration time reaches its maximum or the convergence condition is satisfied, the unknown calibration parameters can be estimated. With ε defined as the convergence threshold, the convergence condition can be represented as follows:

(7) $$\left|e(m_{xx},\ldots ,m_{zz},a_{x\_e},\ldots ,a_{z\_e})\right|<\varepsilon$$

The external interference of a magnetic field can be divided into hard magnetic interference and soft magnetic interference. The hard magnetic interference would cause the measured data to deviate from the sphere origin as a whole. The soft magnetic interference would cause the shape of data distribution to change from a standard sphere to an ellipsoid. The error model of the magnetometer can be represented as follows:

(8) $$\left[\begin{array}{c} m_{x\_c}\\ m_{y\_c}\\ m_{z\_c} \end{array}\right]=\left[\begin{array}{ccc} k_{x} & k_{y} & k_{z} \end{array}\right]\cdot \left[\begin{array}{c} m_{x}\\ m_{y}\\ m_{z} \end{array}\right]+\left[\begin{array}{c} c_{x}\\ c_{y}\\ c_{z} \end{array}\right]$$

where $m_{i}\left(i=x,y,z\right)$ denotes the raw data of the magnetometer; $m_{i\_c}\left(i=x,y,z\right)$ the corrected magnetic field data; $c_{i}\left(i=x,y,z\right)$ the hard magnetic calibration parameters; $k_{i}\left(i=x,y,z\right)$ the soft magnetic calibration parameters. The magnetometer should be rotated in all directions to find out the maximum and minimum values of each axis. The geomagnetic calibration parameters can be calculated as follows:

(9) $$\left\{\begin{array}{c} c_{x}=\frac{X_{max}+X_{min}}{2}\\ c_{y}=\frac{Y_{max}+Y_{min}}{2}\\ c_{z}=\frac{Z_{max}+Z_{min}}{2} \end{array}\right.$$

(10) $$\left\{\begin{array}{c} k_{x}=\frac{X_{max}-X_{min}}{X_{max}-X_{min}}\\ k_{y}=\frac{X_{max}-X_{min}}{Y_{max}-Y_{min}}\\ k_{z}=\frac{X_{max}-X_{min}}{Z_{max}-Z_{min}} \end{array}\right.$$

where $X_{max}$, $X_{min}$, $Y_{max}$, $Y_{min}$, $Z_{max},\;\mathrm{and}\;Z_{min}$ are the maximum and minimum values of three axes.

### 3.2. Sensor Fusion

Several methods are available for representing attitudes in space, such as Euler angle, direction cosine, and quaternion. The direction cosine method can be used to solve matrix transformations, but it requires extensive calculations. The Euler angle method is easy to understand, but it may cause the gimbal lock problem. The quaternion method is more efficient and requires fewer calculations compared to the other two methods. Since the update of the quaternion depends on the previous state, the acceleration and geomagnetism data in the static state is used to calculate the initial quaternion attitude. The initial pitch angle $\theta_{0}$ and roll angle $\gamma_{0}$ can be calculated using the following equation:

(11) $$\left\{\begin{array}{c} \theta_{0}=arcsin\left(a_{y}^{n}\right)\\ \gamma_{0}=-arctan\left(\frac{a_{x}^{n}}{a_{z}^{n}}\right) \end{array}\right.$$

Specifically, ${\left[\begin{array}{ccc} a_{x}^{n} & a_{y}^{n} & a_{z}^{n} \end{array}\right]}^{T}$ is the projection of gravitational acceleration from reference frame to carrier frame. Then, the initial yaw angle can be calculated with $\theta_{0}$, $\gamma_{0}$, and the projection of magnetic field data ${\left[\begin{array}{ccc} m_{x}^{n} & m_{y}^{n} & m_{z}^{n} \end{array}\right]}^{T}$ as follows:

(12) $$\left\{\begin{array}{c} m_{y}^{n}=m_{x}^{b}cos\gamma_{0}+m_{y}^{b}sin\gamma sin\theta_{0}+m_{z}^{b}sin\gamma cos\theta_{0}\\ m_{x}^{n}=m_{y}^{b}cos\theta_{0}-m_{z}^{b}sin\theta_{0}\\ \varphi_{0}=\;arctan\left(\frac{m_{y}^{n}}{m_{x}^{n}}\right) \end{array}\right.$$

When ${\left[\begin{array}{ccc} \theta_{0} & \gamma_{0} & \varphi_{0} \end{array}\right]}^{T}$ is confirmed, the initial quaternion can be calculated using the following equations:

(13) $$\left\{\begin{array}{c} i\text{\_}q_{0}=\cos\left(\frac{\gamma_{0}}{2}\right)\cos\left(\frac{\theta_{0}}{2}\right)\cos\left(\frac{\varphi_{0}}{2}\right)-\sin\left(\frac{\gamma_{0}}{2}\right)\sin\left(\frac{\theta_{0}}{2}\right)\sin\left(\frac{\varphi_{0}}{2}\right)\\ i\text{\_}q_{1}=\cos\left(\frac{\gamma_{0}}{2}\right)\sin\left(\frac{\theta_{0}}{2}\right)\cos\left(\frac{\varphi_{0}}{2}\right)-\sin\left(\frac{\gamma_{0}}{2}\right)\cos\left(\frac{\theta_{0}}{2}\right)\sin\left(\frac{\varphi_{0}}{2}\right)\\ i\text{\_}q_{2}=\sin\left(\frac{\gamma_{0}}{2}\right)\cos\left(\frac{\theta_{0}}{2}\right)\cos\left(\frac{\varphi_{0}}{2}\right)+\cos\left(\frac{\gamma_{0}}{2}\right)\sin\left(\frac{\theta_{0}}{2}\right)\sin\left(\frac{\varphi_{0}}{2}\right)\\ i\text{\_}q_{3}=\cos\left(\frac{\gamma_{0}}{2}\right)\cos\left(\frac{\theta_{0}}{2}\right)\sin\left(\frac{\varphi_{0}}{2}\right)+\sin\left(\frac{\gamma_{0}}{2}\right)\sin\left(\frac{\theta_{0}}{2}\right)\cos\left(\frac{\varphi_{0}}{2}\right) \end{array}\right.$$

**Figure 7:**
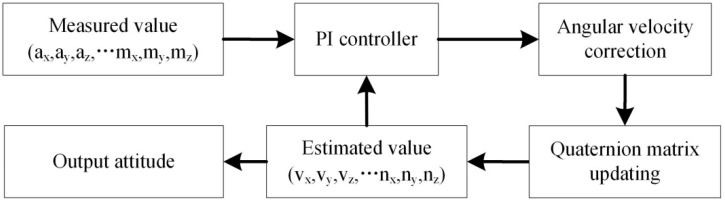
Flowchart of sensor fusion using a complementary filter algorithm.

As proven by the calculation process of the initial Euler angle, the yaw, pitch, and roll angles can be calculated directly from the acceleration, angular velocity, and magnetic field data in the static state. However, when the sensor is in motion, extra linear acceleration is introduced, resulting in inaccurate calculation of the sensor’s attitude. In addition, the integral drift still exists when angular velocity is used to calculate the attitude after sensor calibration. In this paper, a complementary filter algorithm is applied to solve the multi-sensor fusion problem [28,29]. The essence of multi-sensor fusion is to use the proportional–integral (PI) method to calculate the error between the estimated and measured values of acceleration and geomagnetism, and apply this error to correct the updated quaternion. The sensor fusion process is as shown in Figure 7.

${\left[\begin{array}{ccc} e_{ax} & e_{ay} & e_{az} \end{array}\right]}^{T}$ is defined as the error caused by the acceleration data in carrier coordinates; it can be expressed as follows:

(14) $$\left[\begin{array}{c} e_{ax}\\ e_{ay}\\ e_{az} \end{array}\right]=a\times v=\left|\begin{array}{ccc} i & j & k\\ a_{x} & a_{y} & a_{z}\\ v_{x} & v_{y} & v_{z} \end{array}\right|=\left[\begin{array}{c} a_{y}\cdot v_{z}-a_{z}\cdot v_{y}\\ a_{z}\cdot v_{x}-a_{x}\cdot v_{z}\\ a_{x}\cdot v_{y}-a_{z}\cdot v_{x} \end{array}\right]$$

where ${\left[\begin{array}{ccc} v_{x} & v_{y} & v_{z} \end{array}\right]}^{T}$ is the estimated acceleration in carrier coordinates.

${\left[\begin{array}{ccc} e_{mx} & e_{my} & e_{mz} \end{array}\right]}^{T}$ is defined as the error caused by the geomagnetic field data in carrier coordinates; it can be expressed as follows:

(15) $$\left[\begin{array}{c} e_{mx}\\ e_{my}\\ e_{mz} \end{array}\right]=m\times w=\left|\begin{array}{ccc} i & j & k\\ m_{x} & m_{y} & m_{z}\\ w_{x} & w_{y} & w_{z} \end{array}\right|=\left[\begin{array}{c} m_{y}\cdot n_{z}-m_{z}\cdot n_{y}\\ m_{z}\cdot n_{x}-m_{x}\cdot n_{z}\\ m_{x}\cdot n_{y}-m_{z}\cdot n_{x} \end{array}\right]$$

where ${\left[\begin{array}{ccc} n_{x} & n_{y} & n_{z} \end{array}\right]}^{T}$ is the estimated geomagnetic field data in carrier coordinates.

Then, the total error can be defined as:

(16) $$\left[\begin{array}{c} e_{x}\\ e_{y}\\ e_{z} \end{array}\right]=\left[\begin{array}{c} e_{ax}\\ e_{ay}\\ e_{az} \end{array}\right]+\left[\begin{array}{c} e_{mx}\\ e_{my}\\ e_{mz} \end{array}\right]=\left[\begin{array}{c} \left(a_{y}\cdot v_{z}-a_{z}\cdot v_{y}\right)+\left(m_{y}\cdot n_{z}-m_{z}\cdot n_{y}\right)\\ \left(a_{z}\cdot v_{x}-a_{x}\cdot v_{z}\right)+\left(m_{z}\cdot n_{x}-m_{x}\cdot n_{z}\right)\\ \left(a_{x}\cdot v_{y}-a_{z}\cdot v_{x}\right)+\left(m_{x}\cdot n_{y}-m_{z}\cdot n_{x}\right) \end{array}\right]$$

The angular velocity can be fixed using the following equations:

(17) $$\left\{\begin{array}{c} \left[\begin{array}{c} e_{x}i\\ e_{y}i\\ e_{z}i \end{array}\right]=\left[\begin{array}{c} e_{x}i\\ e_{y}i\\ e_{z}i \end{array}\right]+K_{i}\cdot \left[\begin{array}{c} e_{x}\\ e_{y}\\ e_{z} \end{array}\right]\\ \left[\begin{array}{c} \omega_{x}^{\prime }\\ \omega_{y}^{\prime }\\ \omega_{z}^{\prime } \end{array}\right]=\left[\begin{array}{c} \omega_{x}\\ \omega_{y}\\ \omega_{z} \end{array}\right]+K_{p}\cdot \left[\begin{array}{c} e_{x}\\ e_{y}\\ e_{z} \end{array}\right]+\left[\begin{array}{c} e_{x}i\\ e_{y}i\\ e_{z}i \end{array}\right] \end{array}\right.$$

where ${\left[\begin{array}{ccc} \omega_{x} & \omega_{y} & \omega_{z} \end{array}\right]}^{T}$ is the measured angular velocity from a gyroscope, and ${\left[\omega_{x}^{\prime }\;\omega_{y}^{\prime }\;\omega_{z}^{\prime }\right]}^{T}$ is the fixed angular velocity to update the quaternion matrix. The updated quaternion attitude ${\left[\begin{array}{ccc} q_{0}^{\prime } & q_{1}^{\prime }\;\;q_{2}^{\prime } & q_{3}^{\prime } \end{array}\right]}^{T}$ can be represented as follows:

(18) $$\left[\begin{array}{c} q_{0}^{\prime }\\ q_{1}^{\prime }\\ q_{2}^{\prime }\\ q_{3}^{\prime } \end{array}\right]=\left[\begin{array}{c} q_{0}\\ q_{1}\\ q_{2}\\ q_{3} \end{array}\right]+\left[\begin{array}{cccc} 0 & -q_{1}\cdot \omega_{x}^{\prime } & -q_{2}\cdot \omega_{y}^{\prime } & -q_{3}\cdot \omega_{z}^{\prime }\\ q_{0}\cdot \omega_{x}^{\prime } & 0 & q_{2}\cdot \omega_{z}^{\prime } & -q_{3}\cdot \omega_{y}^{\prime }\\ q_{0}\cdot \omega_{y}^{\prime } & -q_{1}\cdot \omega_{z}^{\prime } & 0 & q_{31}\cdot \omega_{x}^{\prime }\\ q_{0}\cdot \omega_{z}^{\prime } & q_{1}\cdot \omega_{y}^{\prime } & -q_{2}\cdot \omega_{x}^{\prime } & 0 \end{array}\right]\cdot \frac{T}{2}$$

Finally, the Euler angle can be obtained using the following equations:

(19) $$\left\{\begin{array}{c} \theta =\arcsin\left(2\left(q_{2}^{\prime }q_{3}^{\prime }+q_{0}^{\prime }q_{1}^{\prime }\right)\right)\\ \gamma =-\arctan\left(\frac{2\left(q_{1}^{\prime }q_{3}^{\prime }-q_{0}^{\prime }q_{2}^{\prime }\right)}{q_{0}^{\prime 2}-q_{1}^{\prime 2}-q_{2}^{\prime 2}+q_{3}^{\prime 2}}\right)\\ \varphi =\arctan\left(\frac{2\left(q_{1}^{\prime }q_{2}^{\prime }-q_{0}^{\prime }q_{3}^{\prime }\right)}{q_{0}^{\prime 2}-q_{1}^{\prime 2}+q_{2}^{\prime 2}+q_{3}^{\prime 2}}\right) \end{array}\right.$$

where θ, γ, and φ are the pitch, roll, and yaw angles of a single IMMU sensor.

## 4. Experimental Results of Sensor Calibration and Fusion 

Since all the IMMU sensors on the data glove are of the same model (MPU-9250), one of these sensors is used to demonstrate the experimental results of sensor calibration and fusion. 

**Figure 8:**
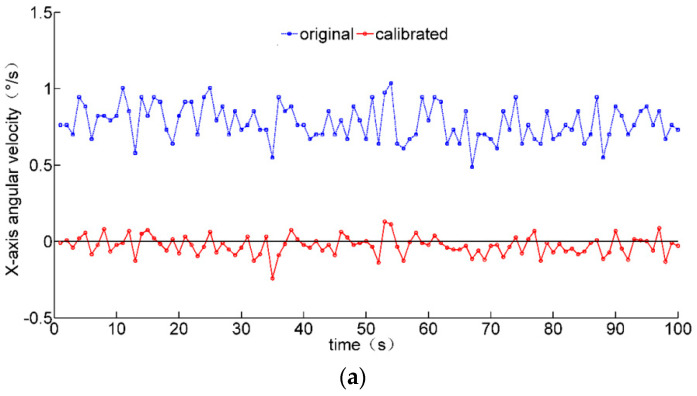

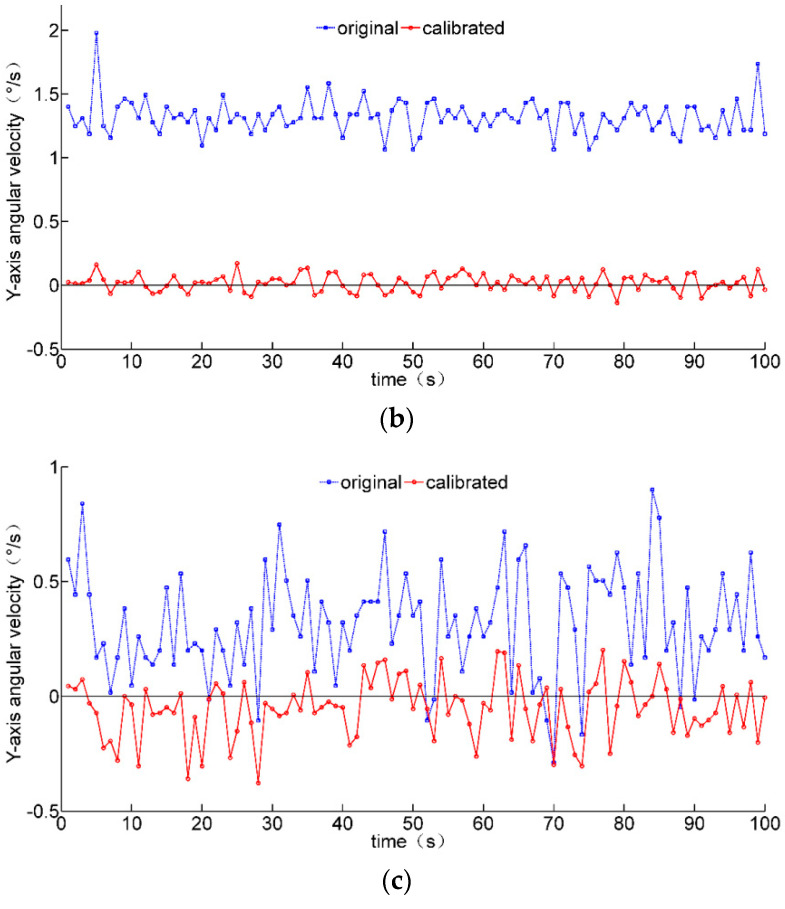
Comparisons of static gyroscope data before and after calibration. (**a**) *X*-axis angular velocity; (**b**) *y*-axis angular velocity; (**c**) *z*-axis angular velocity.

The comparisons of angular velocity data before and after calibration are shown in Figure 8. It can be seen that the zero bias of the gyroscope is reduced. However, the integral drift of the gyroscope still fails to be eliminated after a long period of time, which needs to be corrected by fusing the acceleration and geomagnetism data.

**Figure 9:**
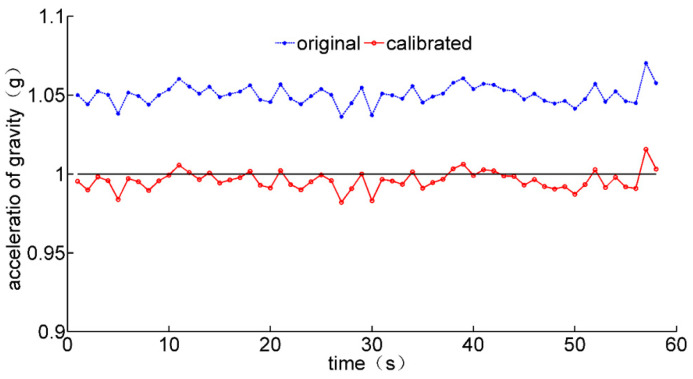
Comparisons of local gravitational acceleration before and after calibration.

The comparisons of normalized acceleration data before and after calibration are shown in Figure 9. The theoretical value of normalized gravitational acceleration in the static state should be equal to 1. The accuracy of local gravitational acceleration is improved after calibration. 

**Figure 10:**
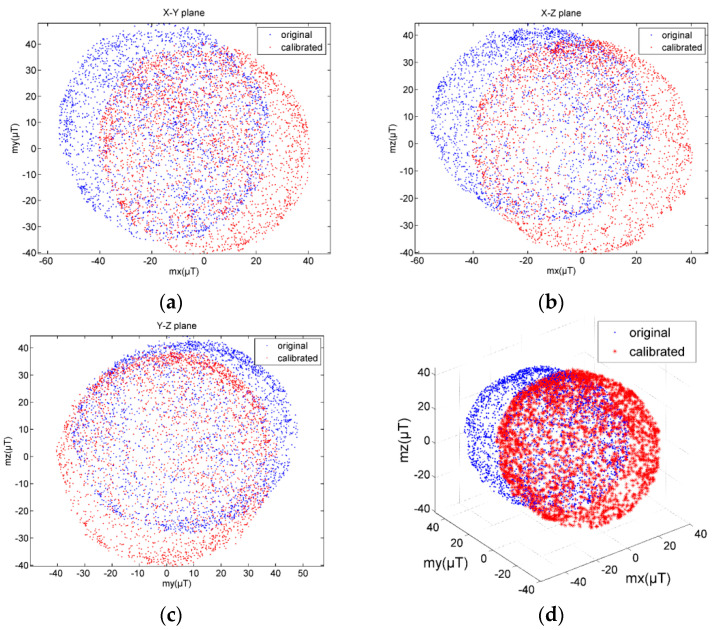
Comparisons of geomagnetic field data distribution before and after calibration. (**a**) X–Y plane; (**b**) X–Z plane; (**c**) Y–Z plane; (**d**) X–Y–Z 3D view.

**Figure 11:**
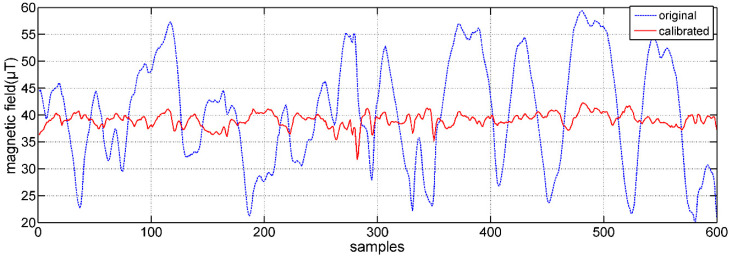
Comparisons of geomagnetic field data before and after calibration.

The geomagnetic field data should lie on the sphere equidistant from the center. The 3D scatter plots of the geomagnetic field data are shown in Figure 10. The comparisons of the geomagnetic field data on X–Y, X–Z, and Y–Z planes are shown in Figure 11. It can be seen that the data are distributed in a nearly elliptical shape before calibration, and in a nearly standard spherical shape after calibration. 

**Figure 12:**
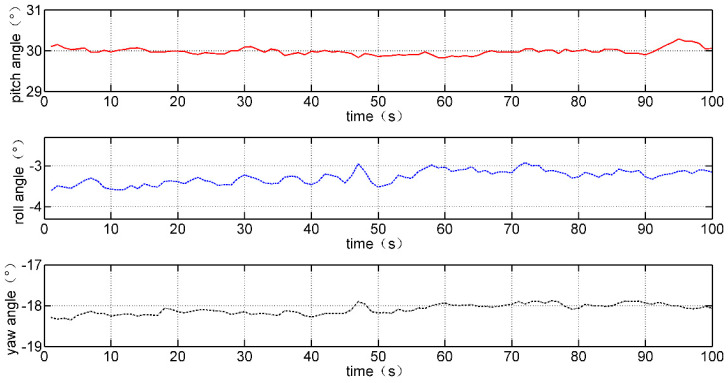
Pitch, roll, and yaw angles in the static state (when pitch angle is set to 30°).

As discussed earlier, the attitude angles of the inertial sensor can be calculated using a complementary filter algorithm. The attitude angles of the IMMU sensor in the static state are shown in Figure 12. The static accuracy of attitude angles is smaller than 1°, which satisfies the application requirement of our data glove system. 

**Figure 13:**
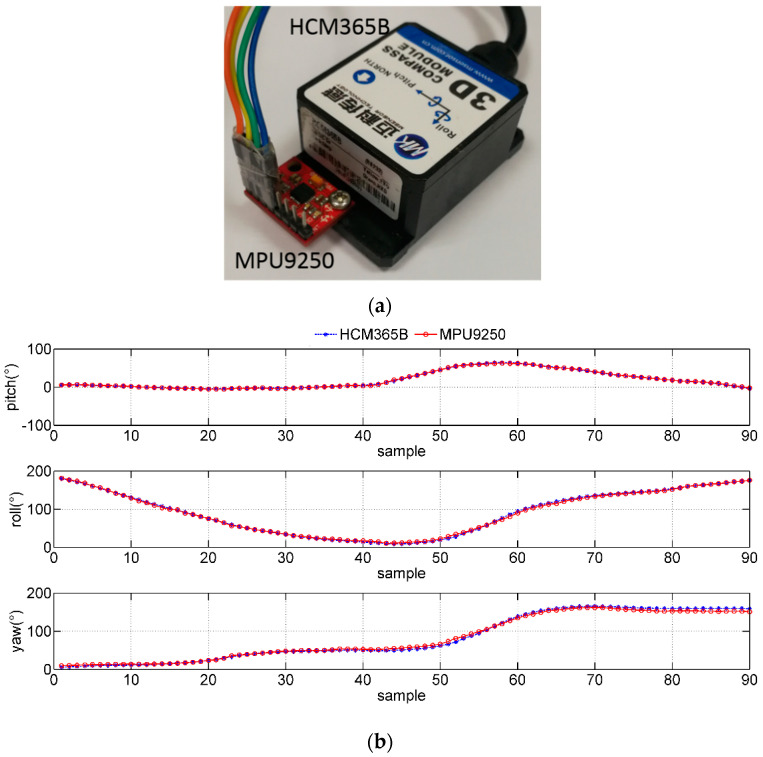
Comparisons of measured dynamic attitude angles between MPU-9250 and HCM365B. (**a**) HCM365B E-compass and MPU-9250 IMMU sensor; (**b**) dynamic attitude angle changes in motion.

The commercial HCM365B E-compass is used to evaluate the dynamic performance of the IMMU sensor, as shown in Figure 13a. The output attitude angle curves of HCM365B and the IMMU sensor are very close, while the roll angle changes from 0° to 200°.

## 5. Human–Computer Interaction Using Data Glove

**Figure 14:**
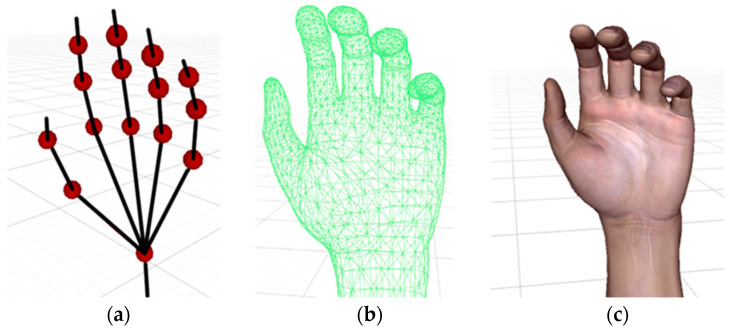
Human hand modeling in the Unity platform. (**a**) Hierarchy of joints; (**b**) hand skinned mesh; (**c**) virtual hand model.

Real-time human–computer interaction is also a required function for patients while doing rehabilitation exercises at home. A series of virtual scenes needs to be established by the human–computer interaction module, including a virtual hand, movable virtual items, lights, and a background. After the virtual scenes are built, all the 12 IMMU sensors will be mapped to the joints of the virtual hand by converting sensor attitude angles into finger rotation angles. In this paper, a virtual hand is established in the Unity platform, as shown in Figure 14.

**Figure 15:**
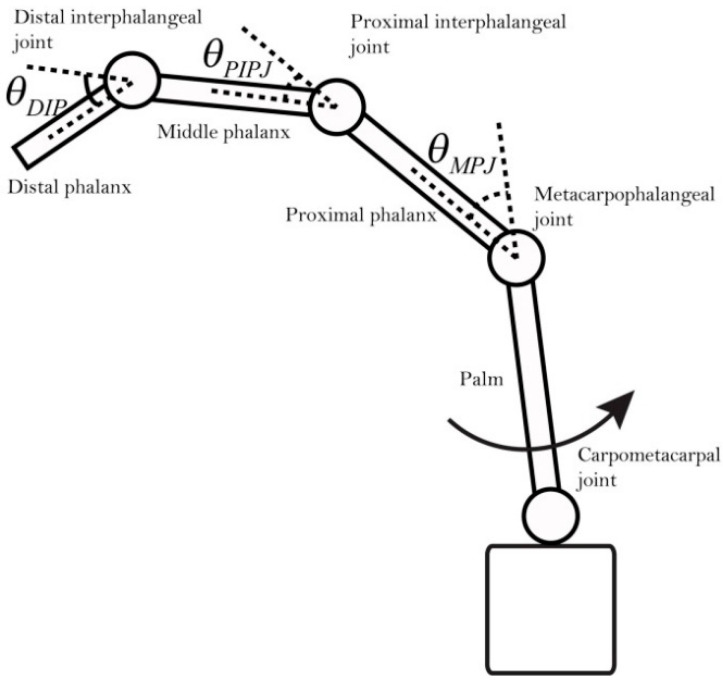
Schematic side view of the bending angles of different joints.

The human interaction module includes the following three parts: (1) Virtual scene design: The virtual hand should be established according to the skeletal structure of the human hand. Some virtual items are also provided for practicing hand grasping motions. (2) Sensor–joints mapping: 12 IMMU sensors need to be mapped to the corresponding joints. (3) Scripts: The scripts include finding joint objects in the hierarchical view, mapping sensors to model joints, receiving attitude angles, and displaying animations. The bending angles of the joints of the virtual hand are limited to a range that fits in with daily human hand motions. A schematic diagram showing the bending angles of different joints is provided in Figure 15. 

**Figure 16:**
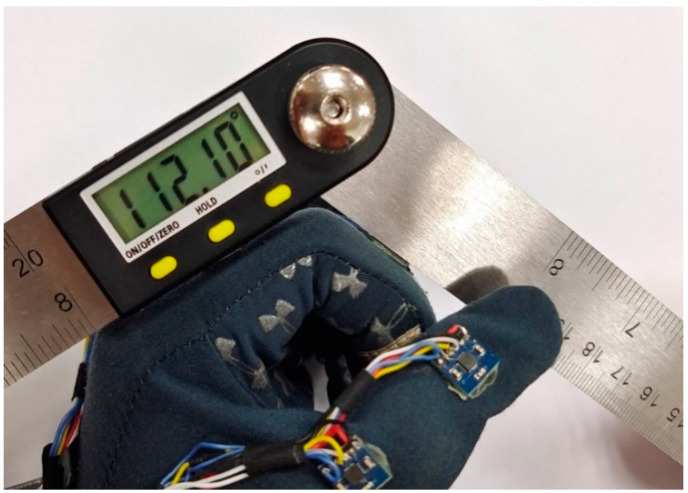
Comparison of the measured angle between data glove and digital goniometer.

To validate the joint angle obtained by the data glove, the digital goniometer is used to measure the angle, as shown in Figure 16. One problem of using such a steel goniometer is that the magnetometer inside the IMMU sensor may be disturbed by nearby ferromagnetic materials. Therefore, the digital goniometer should be placed on the finger after the angle data have been exported from the data glove. 

The comparison of measurement results is represented in Table 1. The angles measured by goniometer and data glove are recorded when the index finger is held in four different positions. The average error rate is less than 2% and the maximum deviation is nearly 1.4°. The accuracy of the data glove will be enough for most wearable applications.

Hand rehabilitation assessment, by the total active range of motion (TAM) method, for example, requires the bending angle data of the joints, which can be calculated with the attitude angles of sensors on the data glove. Since the DIP joint has no sensor placed on it, its bending angle is empirically set to 1/3 that of the PIP joint. The relationship between the bending angles of joints and the attitude angles of sensors is represented in Table 2.

**Figure 17:**
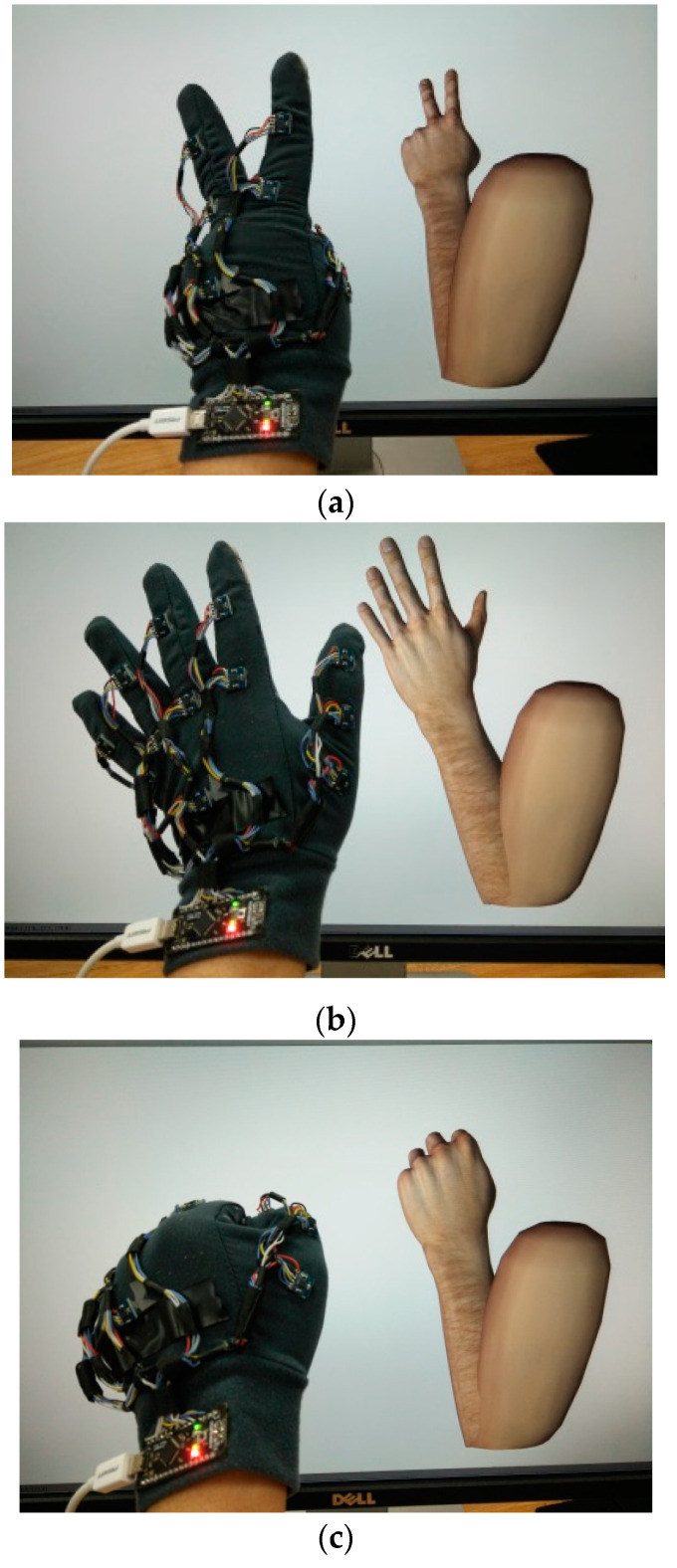
Gestures reproduced by the virtual hand in Unity. (**a**) Gesture “2”; (**b**) gesture “5”; (**c**) gesture “10”.

Specifically, $S_{i}\left(i=1,\;2,\;\cdots ,\;12\right)$ represents the attitude angles of IMMU sensors around the *x*-axis. The experimental results of three hand gestures showing the numbers ”2”, “5”, and “10” are provided in Figure 17. The hand gestures are captured and reconstructed with the virtual hand in Unity with high accuracy. The gesture data can be saved and exported by physicians for off-line diagnosis, as shown in Table 3.

**Figure 18:**
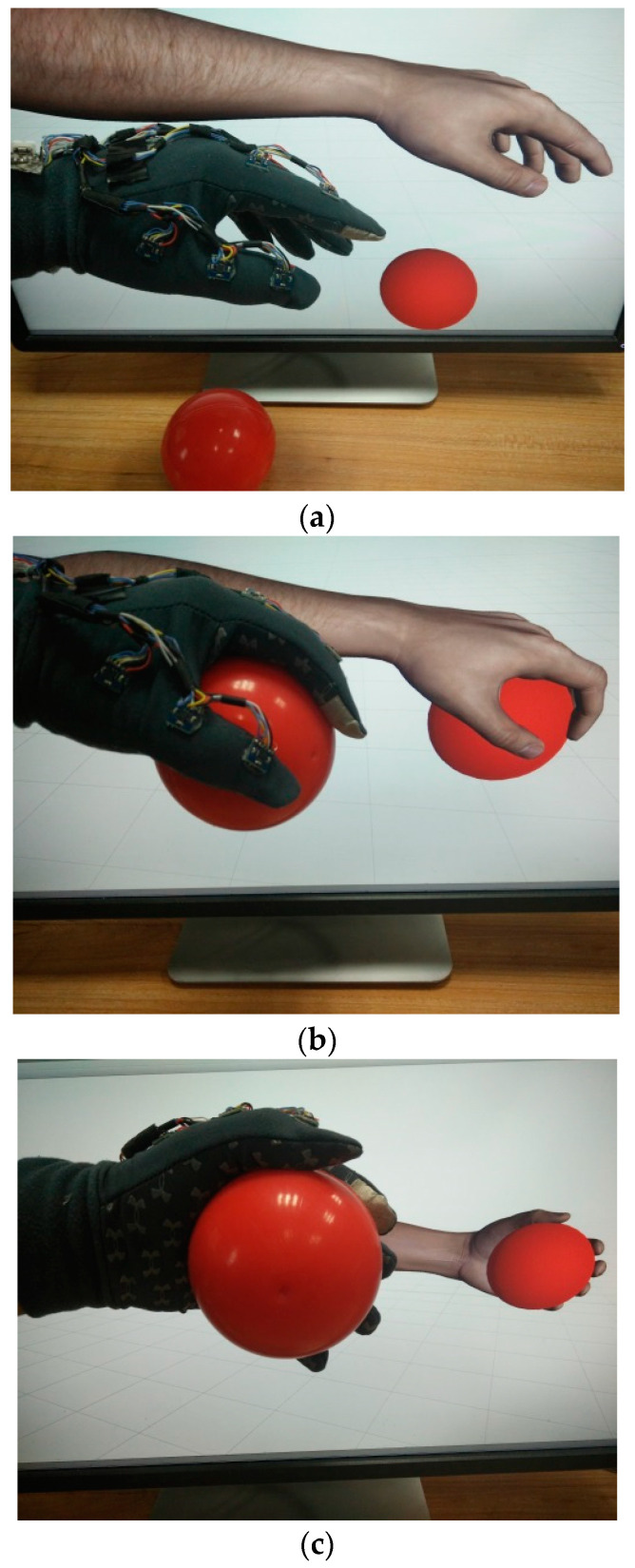
Interactions with a ball using the proposed data glove. (**a**) Locating the ball; (**b**) grasping the ball; (**c**) flipping the hand.

**Figure 19:**
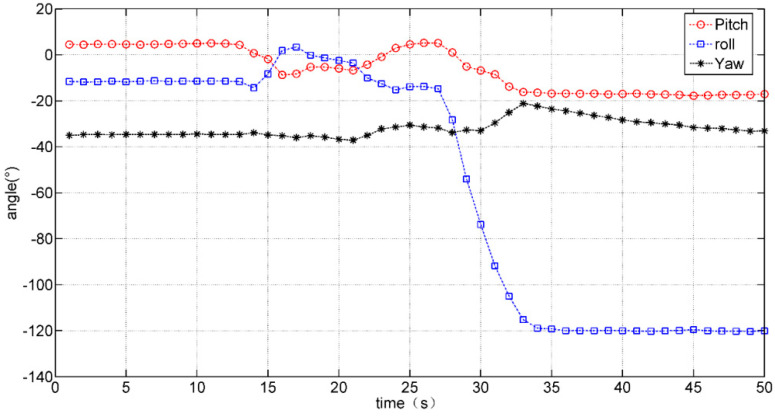
Hand posture changes during ball grasping experiment.

The dynamic ball grasping experiment is demonstrated in Figure 18. The virtual ball and hand should be set in such original positions that their distance is the same as that between the physical ball and hand. The subject needs to pick up the ball and flip the hand several times. The changes in hand posture can be reviewed from the data of Sensor 8 on the back of the hand, as shown in Figure 19. The recovery status can be estimated from the recorded data, for example, the time taken for an action, the accuracy of an action, and the degree of hand tremor.

Four different data glove systems are compared with our proposed IMMU data glove, as listed in Table 4. It can be found that both the optical fiber sensor-based glove system and IMMU sensor-based data glove system can provide a good performance while measuring the joint angle. However, the cost of IMMU sensors is much less than optical fiber sensors for massive commercial wearable applications. 

## 6. Conclusions

This paper presents a novel data glove system to assess hand function during hand rehabilitation. 12 nine-axis inertial sensors are integrated in a data glove to obtain the angles of hand joints. The error models of the accelerometer, gyroscope, and magnetometer are analyzed. The least square principle is used to solve the correction parameters of the accelerometer. The validity of the calibration process is verified by comparing raw sensor data and calibrated data. A quaternion-based complementary filtering algorithm is applied to fuse the acceleration, angular velocity and geomagnetism data. A commercial E-compass is also used to verify the stability and dynamic performance of our fusion algorithm. 

Real-time and high-precision human–computer interaction is realized with the data glove and Unity. A virtual hand model, a ball model, and some virtual scenes are established in Unity, and then the attitude angles of 12 sensors are transformed into the rotation angles of hand joints. The experimental results show that the glove system can effectively measure hand postures and joint angles in motion, and it is expected to be used to develop a variety of rehabilitation scenarios to get patients more engaged in the long-term rehabilitation process.

## Figures and Tables

**Table 1:** Measurement results of goniometer and data glove.

Angle from goniometer	112.10°	91.20°	80.52°	69.65°
Average angle from data glove	110.28°	89.55°	79.66°	68.30°
Error rate	1.6%	1.8%	1.1%	1.9%

**Table 2:** Calculation of bending angles of joints using sensor attitudes.

Joint	ID	Bending Angle
Thumb IP joint	$\theta_{\mathrm{IP}\_\mathrm{Thumb}}$	$S_{1}-S_{2}$
Thumb MP joint	$\theta_{\mathrm{MP}\_\mathrm{Thumb}}$	$S_{3}-S_{8}$
Index finger PIP joint	$\theta_{\mathrm{PIP}\_\mathrm{Index}}$	$S_{4}-S_{5}$
Index finger MP joint	$\theta_{\mathrm{MP}\_\mathrm{Index}}$	$S_{5}-S_{8}$
Middle finger PIP joint	$\theta_{\mathrm{PIP}\_\mathrm{Middle}}$	$S_{6}-S_{7}$
Middle finger MP joint	$\theta_{\mathrm{MP}\_\mathrm{Middle}}$	$S_{7}-S_{8}$
Ring finger PIP joint	$\theta_{\mathrm{PIP}\_\mathrm{Ring}}$	$S_{9}-S_{10}$
Ring finger MP joint	$\theta_{\mathrm{MP}\_\mathrm{Ring}}$	$S_{10}-S_{8}$
Little finger PIP joint	$\theta_{\mathrm{PIP}\_\mathrm{Little}}$	$S_{11}-S_{12}$
Little finger MP joint	$\theta_{\mathrm{MP}\_\mathrm{Little}}$	$S_{12}-S_{8}$

**Table 3:** Bending angles of joints with three gestures.

Gesture	Index Finger		Middle Finger		Ring Finger		Little Finger	
	$\theta_{\mathrm{PIPJ}}$	$\theta_{\mathrm{MPJ}}$	$\theta_{\mathrm{PIPJ}}$	$\theta_{\mathrm{MPJ}}$	$\theta_{\mathrm{PIPJ}}$	$\theta_{\mathrm{MPJ}}$	$\theta_{\mathrm{PIPJ}}$	$\theta_{\mathrm{MPJ}}$
“2”	9.2°	7.9°	2.4°	24.8°	109.2°	49.4°	64.4°	90.7°
“5”	5.5°	2.2°	16.4°	3.9°	9.5°	10.5°	24.3°	20.6°
“10”	110.3°	70.2°	108.2°	67.5°	148.6°	62.9°	68.9°	77.3°

**Table 4:** Comparison of proposed data glove and other data glove systems.

Publications	Type of Sensor	Number of Sensors	Deviation of Joint Angle
Cha et al. [30]	flexible piezoelecric sensor	19 (one hand)	5°
da Silva et al. [21]	fiber bragg gratings sensor	1 (each finger)	2°
Li et al. [31]	optical linear encoder	3 (each finger)	1°
Kortier et al. [32]	IMMU	3 (each finger)	1.1°
Proposed glove system	IMMU	12 (one hand)	1.4°

## Data Availability

The data presented in this study are available in the article.
